# The Discovery of Putative Urine Markers for the Specific Detection of Prostate Tumor by Integrative Mining of Public Genomic Profiles

**DOI:** 10.1371/journal.pone.0028552

**Published:** 2011-12-16

**Authors:** Min Chen, Kai Wang, Liang Zhang, Cheng Li, Yongliang Yang

**Affiliations:** 1 Center for Molecular Medicine, School of Life Science and Biotechnology, Dalian University of Technology, Dalian, People's Republic of China; 2 Department of Biostatistics and Computational Biology, Dana-Farber Cancer Institute, Harvard School of Public Health, Boston, Massachusetts, United States of America; 3 School of Software, Dalian University of Technology, Dalian, People's Republic of China; Roswell Park Cancer Institute, United States of America

## Abstract

Urine has emerged as an attractive biofluid for the noninvasive detection of prostate cancer (PCa). There is a strong imperative to discover candidate urinary markers for the clinical diagnosis and prognosis of PCa. The rising flood of various omics profiles presents immense opportunities for the identification of prospective biomarkers. Here we present a simple and efficient strategy to derive candidate urine markers for prostate tumor by mining cancer genomic profiles from public databases. Prostate, bladder and kidney are three major tissues from which cellular matters could be released into urine. To identify urinary markers specific for PCa, upregulated entities that might be shed in exosomes of bladder cancer and kidney cancer are first excluded. Through the ontology-based filtering and further assessment, a reduced list of 19 entities encoding urinary proteins was derived as putative PCa markers. Among them, we have found 10 entities closely associated with the process of tumor cell growth and development by pathway enrichment analysis. Further, using the 10 entities as seeds, we have constructed a protein-protein interaction (PPI) subnetwork and suggested a few urine markers as preferred prognostic markers to monitor the invasion and progression of PCa. Our approach is amenable to discover and prioritize potential markers present in a variety of body fluids for a spectrum of human diseases.

## Introduction

Prostate cancer (PCa) remains to be the most common malignancy and the second cause of cancer-related death for men worldwide [1]. Particularly in the western world, the number of men diagnosed with PCa has increased by 30% over the last 25 years and is expected to be doubled by the year of 2030 [2]. PCa is generally curable when the primary lesion is within its benign state but very difficult to cure or no longer curable once the tumor has spread to other distant sites. Therefore, the early detection is essential for the successful clinical treatment of PCa. Currently, the combination of DRE (digital rectal exam) and the PSA (prostate-specific antigen) blood test is commonly used in screening test to detect PCa in the absence of symptoms. Unfortunately, it is well recognized that the usefulness of PSA suffers from its low specificity and its low positive predictive value in early PCa detection. For example, it has been found that the upper cut-off of the PSA reference level at 4.0 ng/ml fails to detect a large number of PCa and many men with PSA values <4.0 ng/ml actually have PCa [3]. Moreover, it has been demonstrated that PSA can be secreted from other cancerous cells into the bloodstream as well [4]. Hence, there is a clear need to identify putative molecular signatures that can facilitate the accurate and non-invasive clinical PCa detection.

Urine represents an amenable and appealing body fluid for the early detection of PCa [5]. First, urine can be used to detect the presence of PCa because secreted prostatic products or exfoliated cancerous cells are released directly into the genitourinary tract. Second, urine can be easily collected in large amounts noninvasively and repeatedly, rendering it as an attractive material for the analysis of prostate malignancy. To date, a number of urine biomarkers such as *GSTP-1* (glutathione-S-transferase P1), *DD3* (prostate cancer antigen 3, PCA3) and *TB-15* (thymosin β15) etc. have been proposed as potential diagnostic agents for early PCa detection [6]. Moreover, with the recently developed sophisticated mass-spectrometry (MS) technology, it becomes possible to detect certain endogenous metabolites in urine for the early diagnosis of PCa. For instance, Sreekumar et al. [7] have identified Sarcosine (N-methylglycine) as a key metabolite in urine that could be potentially used as a marker for PCa malignancy. Although promising, there are still few studies assessing urine markers for PCa detection and there are only a few candidate urine markers are under consideration for future clinical development. Further, no single marker is adequate for the accurate detection of PCa owing to the complexity and heterogeneity of the disease. Hence, it is clear that a panel of urine markers is required for the successful diagnosis of PCa.

The explosion of biological data and information generated from high-throughput ‘Omics’ technologies such as microarrays has provided unprecedented opportunities for researchers to uncover biomarkers and phenotypic pathways of clinical importance [8]. For instance, Kim *et al.* have reported the mining of public gene profiles from CGAP and GEO database to identify seven putative markers for lung cancer [9]. Analogously, we have successfully identified lists of blood-borne markers for six common human cancer types through a combined mining strategy in the Oncomine microarray database and a pathway knowledgebase. Using a filter-based approach and comparison analysis, we have retrieved disease-specific blood-based markers for each of the tumor types and common markers shared between different tumors. Notably, a large portion of the retrieved genomic-based markers have been literature-confirmed to be associated with the phenotypic pathways of tumor progression and invasiveness. Such findings would certainly be very useful to delineate potential targets with regards to the diagnosis, prognosis and pathogenesis of human solid tumors.

**Figure 1:**
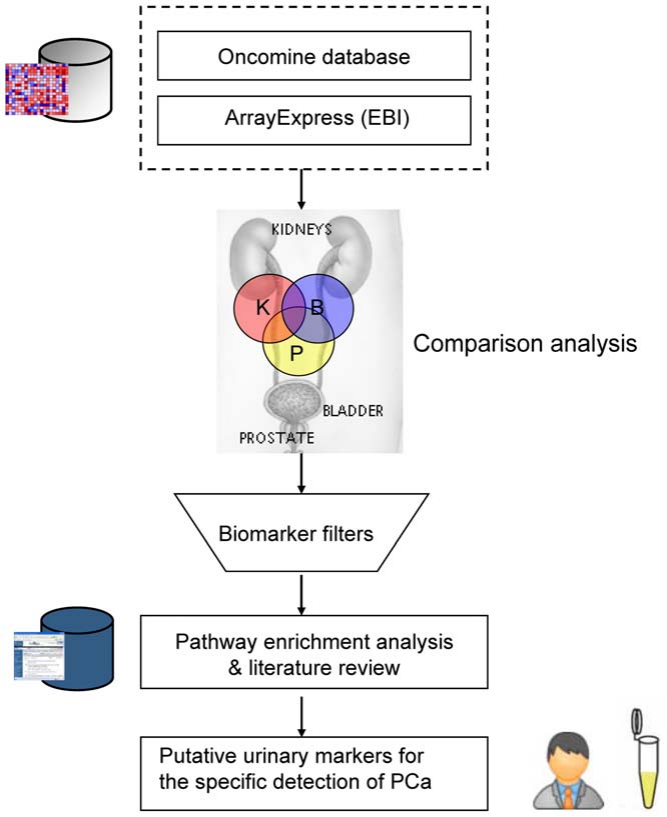
Workflow of integrative mining from public cancer genomic profiles for discovery of putative urinary marker for the specific detection of PCa. In the comparison pie graphs, “B” represents for bladder, “K” represents for kidney and “P” represents for prostate.

Here we present an integrative mining approach to analyze public genomic profiles for the discovery of potential urine markers for PCa detection. Our strategy has been developed in the way that a vast body of cancer genomic profiles can be analyzed in the context of other biological data such as gene ontology, metabolic pathways and gene-gene/protein-protein interaction (PPI) networks (see **Figure 1**). To identify disease-specific markers for PCa, we have retrieved upregulated genes in PCa, bladder cancer and kidney cancer from public cancer genomic databases. We were mining for upregulated genes as PCa markers here mainly because one of the prevailing hypotheses is that the most promising biomarkers for clinical use will be those upregulated genes or their protein products. However, we recognize that this might not be generally true and thereby we don't rule out the possibility that downregulated genes could be interesting candidate markers too. Other researchers could choose to mine downregulated genes for their specific purpose by applying the similar strategy as in this work. These upregulated genes were then filtered through a collection of ontology terms indicating the presence in urine and Ingenuity Knowledgebase. A comparison analysis was performed across prostate, bladder and kidney and only those entities unique to prostate were kept in the list as potential urinary markers for PCa. This is because entities present in bladder cancer and kidney cancer may interfere with the detection of PCa shed in human urinary system. Finally, the putative urine markers for PCa were analyzed and prioritized within metabolic pathways and protein-protein interaction networks. Our strategy highlights the significance of combining a variety of biological data to derive putative markers present in body fluids with disease specificity to detect common and lethal types of human cancers.

## Materials and Methods

The focus of our analysis approach is to retrieve putative markers present in urine for the specific detection of PCa. Therefore, we need to retrieve and filter genes significantly upregulated in PCa, encoding urinary proteins, to a manageable gene list. The choice of microarray platform or database, statistical cut-off criteria, and controlled ontology terms (Gene Ontology terms) in the mining strategy is variable, depending on the particular interest and requirement of the user.

### Microarray data preparation and analysis

In brief, for each of the three tumor types (PCa, bladder cancer and renal cancer), MeSH terms (prostate cancer, prostatic cancer; bladder cancer; kidney cancer, renal cancer) were used to search and obtain microarray experiments characterizing these disease conditions from two popular cancer genomic databases, Oncomine database [10] and ArrayExpress database [11]. Oncomine and ArrayExpress were chosen because they are two of the largest public cancer microarray repositories. Particularly, Oncomine has incorporated 534 independent microarray datasets, which span 35 cancer types. It unifies a large compendium of other published cancer microarray data as well including Gene Expression Omnibus (GEO) and Stanford Microarray Database (SMD). ArrayExpress stores well-annotated raw and normalized cancer microarray data from more than 300 studies. The advantage of using Oncomine and ArrayExpress is that medical researchers could easily perform differential expression analyses comparing most major types of cancer with their respective normal or benign tissues. Those microarray experiments comparing cancer vs. normal including malignant vs. benign conditions measured in equivalent tissues in same experiments were retained. We have chosen a relative stringent FDR (false discovery rate) value cut-off of 0.05 [12] in the analysis process, and only those overexpressed genes with FDR value less than 0.05 are kept in the final list. Overexpressed genes in Oncomine and ArrayExpress were collected by using the same FDR cut-off value. In addition, a customary fold change threshold 2.0 was also applied to retain those significantly overexpressed genes in the list. The redundant genes were resolved from the list. By comparison analysis across the upregulated genes of three tumor types using a C# program (see **File S1**), only those genes specifically upregulated in PCa were retrieved for further analysis.

### Functional annotation enrichment and biomarker filtering

**Figure 2:**
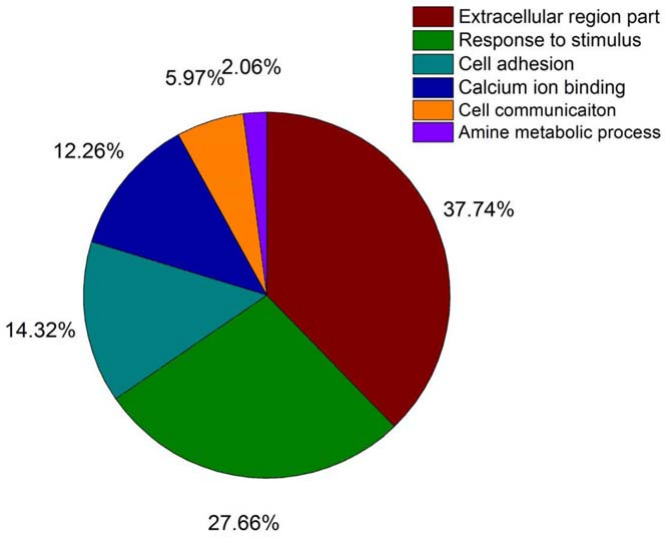
Pie-chart of GO term appearing frequencies among the urinary proteins by clustering analysis of 1273 urinary proteins performed within DAVID system.

Functional annotation (Gene Ontology assignment) for the retrieved overexpressed genes was conducted by using the DAVID system [13]. Next, a set of controlled GO terms implying the presence in urinary proteome were chosen according to the GO clustering analysis of 1273 urinary proteins (see **Figure 2**) collected from MAPU urinary proteome database [14]. The GO clustering analysis was performed within DAVID system and could be used to measure the GO term appearing frequencies among the urinary proteins. Specifically, these controlled GO terms and their appearing frequencies are: Extracelluar region part: 34.8%; Response to stimulus: 25.5%; Cell adhesion: 13.2%; Calcium ion binding: 11.3%; Cell communication: 5.5%; Amine metabolic process: 1.9%. These controlled GO terms are enriched and overrepresented in the urinary proteome through the study of GO clustering. Further, the retrieved putative urine markers were consulted in Sys-BodyFluid database [15], MAPU proteome database and Ingenuity Knowledgebase [16] to confirm their presence in urine. Entities that are not present in urine were removed from the list. These databases represent the three most comprehensive public body fluid proteomes and contain over 10,000 proteins with detailed annotations. Researchers could easily download and analyze protein targets present in various body fluids from these on-line databases.

### Pathway enrichment analysis

The derived list of putative urine markers was then subjected to pathway enrichment analysis by importing them to a few PPI (protein-protein interaction) databases including Pfam [17], InterPro [18], Ingenuity Knowledgebase and the KEGG Knowledgebase [19]. These PPI databases were chosen because they are widely used as reference knowledgebase towards practical applications with network or pathway-based views of proteins, diseases and drugs. Moreover, the millions of pathway interactions storing in these knowledgebase were acquired by curation of scientific publications covering information on genes or proteins. The 19 entities were first imported as seeds to identify overrepresented biological functions and signaling pathways. Entities with direct physical interactions and co-expression evidenced by literatures were identified and used to construct PPI network. Particularly, those entities associated with the tumor cell growth, development and proliferation were used as seeds to construct a PPI subnetwork related to the invasion and metastasis of PCa. Subnetworks were constructed such that the genes (proteins) were nodes, with edges between genes indicating the direction and indirect biological interactions between entities.

### Literature Review of the Candidate Entities

Further analysis and assessment of the resulting putative markers was performed retrospectively using GeneCards (www.genecards.org), a curated database that finds links and cited articles to genes/proteins. The entities obtained were checked by carefully reading the associated literature references or original publications. The accuracy of the findings is assessed using control entities, selected as candidate molecules by other studies or well-known and clinically useful targets for PCa.

## Results

**Figure 3:**
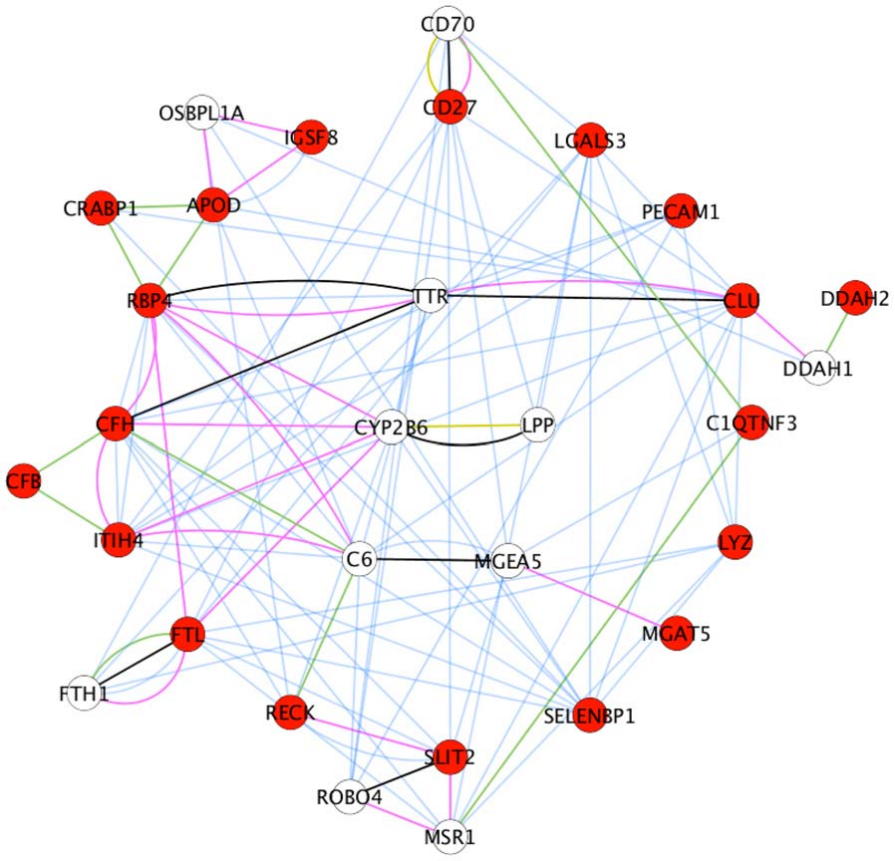
Identification of gene network consisting of 19 putative urine markers (Cytoscape/Genemania). Nineteen putative marker genes are represented as red nodes and the other highly relevant genes are in white. Co-expressed genes are linked by blue lines, genes with same protein domains are linked by green lines, co-localization relationships are described as pink lines, and physical interaction connections are linked by black lines.

**Figure 4:**
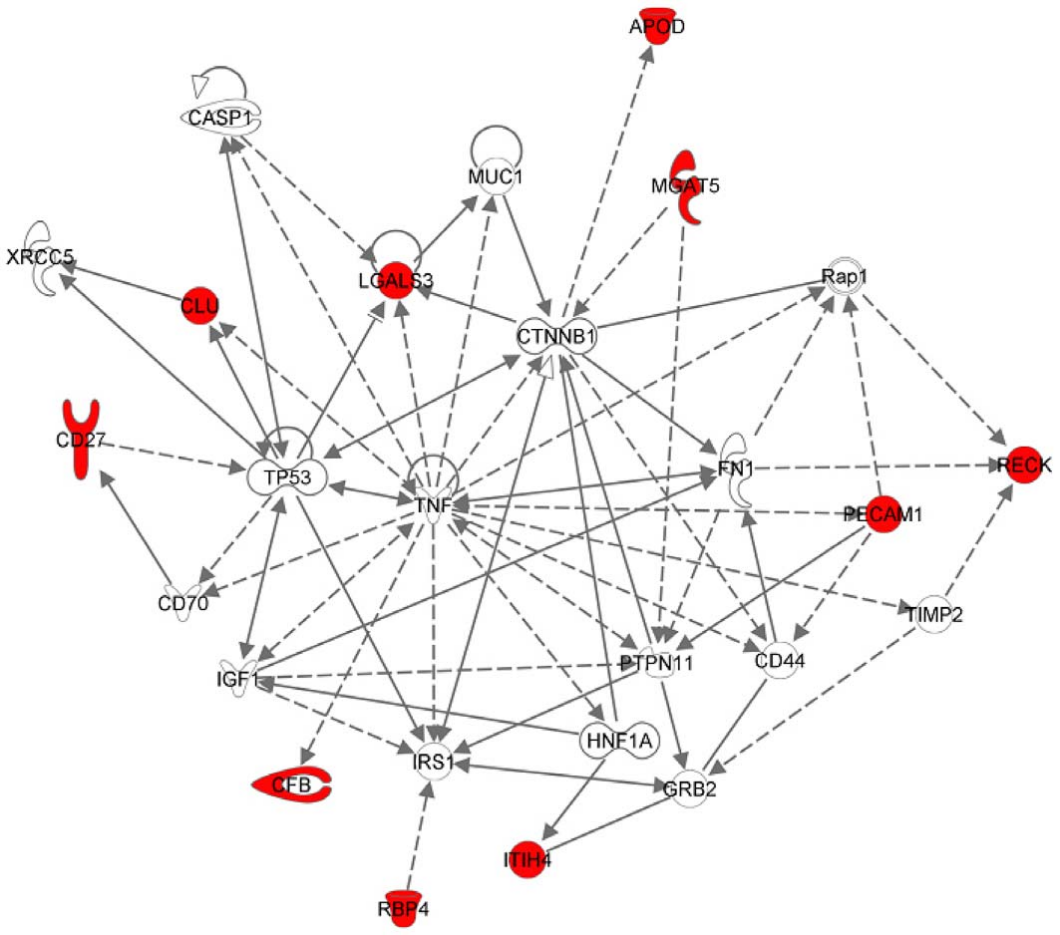
Identification of ‘focused’ protein-protein interaction (PPI) network that may lead to PCa progression and metastasis. Gene products are represented as nodes and biological relationships (direct and indirect) are described as lines (protein–protein interactions) and dashed lines (regulations of bindings, inhibitions, proteolysis, phosphorylation or modifications). The hub entities that are synergistically regulated in this subnetwork are highlighted as red color including *CD27*, *CLU*, *LGALS3*, *APOD*, *MGAT5*, *CFB*, *RBP4*, *ITIH4*, *PECAM1* and *RECK*. Their subcellular locations are not indicated in the figure.

The integrative mining approach assembling Oncomine, ArrayExpress and GEO databases has yielded 5 microarray datasets for bladder, 8 microarray datasets for renal and 15 microarray datasets for prostate (cancer vs. normal including malignant vs. benign condition) by using specific MeSH terms for each tissue type. The mining of these datasets by a relative stringent FDR value cut-off of 0.05 has yielded between 1,112 (renal), 11,191 (bladder) and 13,595 (prostate) overexpressed genes for the three cancer types. Next, a comparison analysis across the three tumor types has yielded a list of 3964 uniquely upregulated genes in PCa, a list of 2364 uniquely upregulated genes in bladder and a list of 51 uniquely upregulated genes in renal. Sequentially, the 3964 uniquely upregulated genes in PCa were filtered by the seven controlled GO terms to yield a list of 19 putative markers which were further consulted by Sys-BodyFluid database and MAPU proteome database to assess their presence in urine. Finally, the list of 19 urinary proteins were subjected to pathway enrichment analysis within a few most popular PPI databases including Pfam, InterPro, Ingenuity Knowledgebase and KEGG. All the 19 entities were found to be connected as a network together with another 10 entities based on co-expression, shared protein domains, co-localization and protein physical interaction relationships [20], [21] (see **Figure 3**). Sharing a protein domain implies that two entities may have very similar functions, but doesn't guarantee that the two entities are connected in the same pathway; co-expression implies that two entities share similar expression pattern; co-localization implies that two entities are expressed in the same tissues or identified in the same cellular location. Within the network, we have found that RBP4 (retinol binding protein 4, plasma), CFH (complement factor H), ITIH4 (inter-alpha (globulin) inhibitor H4) and FTL (ferritin, light polypeptide) are linked due to co-localization; APOD (apolipoprotein D), RBP4 and CRABP1 (cellular retinoic acid binding protein 1) are found to share protein domains according to INTERPRO and PFAM databases. In addition, we have found that CYP2B6 (cytochrome P450, family 2, subfamily B, polypeptide 6) connects with four putative markers because of co-localization; C6 (complement component 6) connects to RBP4 and ITIH4 by co-localization; C6 connects to CFH and RECK (reversion-inducing-cysteine-rich protein with kazal motifs) by sharing same protein domains; TTR (transthyretin) has physical interaction with RBP4, CFH and CLU (clusterin) genes; OSBPL1A (oxysterol binding protein-like 1A) co-localizes with APOD and IGSF8 (immunoglobulin superfamily, member 8); CD70 (CD70 molecule) connects with the candidate marker CD27 (CD27 molecule) by co-localization and physical interaction; CD70 shares the same protein domain with C1QTNF3 (C1q and tumor necrosis factor related protein 3). Furthermore, we have found these marker proteins are largely associated with a few metabolic pathways such as N-glycan biosynthesis, acute phase response signaling and xenobiotic metabolism signaling etc. Interestingly, we have found that 10 out the 19 urinary markers are closely associated with tumor cell development, growth and proliferation pathway (see **Figure 4**).

### Identification of Putative Urine Markers for PCa Detection

As positive controls, we have highlighted below a few very promising urine marker derived from our study that have also been evidenced by precedent literatures as urinary targets. The five ‘positive control’ entities were chosen according to their fold change values in the order of *LGALS3* (lectin, galactoside-binding, soluble 3), *CFB* (complement factor B), *APOD*, *RECK* and *PECAM1* (platelet/endothelial cell adhesion molecule). These entities stand a good chance of being clinically useful markers as they are most upregulated and their protein products are likely to be overproduced in cancerous cells. We want to remind the reader that the study was strived to identify putative urine markers from genomic profiles thereby it is not sufficient to state that: 1) the encoding product of the gene is present or truly upregulated at the protein level; 2) it is really localized at the desired location (excreted into urine) or normally functioning. Hence, the targets derived from the genomic profiling studies need to be further validated at the protein level through various experimental approaches.

### Lectin, galactoside-binding, soluble, 3 (*LGALS3*/*GAL3*)

One interesting putative urine marker that we have retrieved from the mining study is *LGALS3*, which encodes a member of the galectin family of carbohydrate binding proteins. This protein has been implicated in numerous cellular functions including cell proliferation, apoptosis, angiogenesis, tumor progression and metastasis. In fact, a recent study [22] has suggested that *LGALS3* encoding protein, galectin-3, is cleaved during the progression of PCa and might be associated with the progression and metastasis of PCa cells; Sardana et al [23] have suggested galectin-3 as one of the candidate marker proteins shed and secreted by prostate tumor cells. Remarkably, we have found that *LGALS3* has the largest fold change value of 4.121 in cancerous condition compared to normal condition (see **Table 1**) among the 19 entities, rendering it a highly interesting molecule for the diagnosis and prognosis of PCa.

**Table 1:** Identified urinary markers for the unique detection of prostate tumor.

Symbol	Gene name	Location	Family	Urine	Blood	Fold-change	P value
APOD	apolipoprotein D	Extracellular space	transporter	•	•	2.803	2.00E-03
C1QTNF3	C1q and tumor necrosis factor related protein 3	Extracellular Space	other	•		1.100	2.50E-02
CD27	CD27 molecule	Plasma Membrane	Transmembrane receptor	•	•	1.183	8.19E-04
CFB	complement factor B	Extracellular Space	peptidase	•		3.231	4.00E-03
CFH	complement factor H	Extracellular Space	other	•	•	1.381	1.00E-03
CLU	clusterin	Extracellular Space	other	•		1.638	1.50E-02
CRABP1	cellular retinoic acid binding protein 1	Cytoplasm	transporter	•		1.477	1.20E-02
DDAH2	dimethylarginine dimethylaminohydrolase 2	Cytoplasm	enzyme	•		1.152	4.80E-02
FTL	ferritin, light polypeptide	Cytoplasm	other	•		1.718	3.00E-03
IGSF8	immunoglobulin superfamily, member 8	Plasma Membrane	other	•	•	1.358	6.00E-03
ITIH4	inter-alpha (globulin) inhibitor H4	Extracellular Space	other	•	•	1.215	2.40E-02
LGALS3	lectin, galactoside-binding, soluble, 3	Extracellular Space	other	•	•	4.121	5.90E-04
LYZ	lysozyme	Extracellular Space	enzyme	•	•	2.093	4.00E-03
MGAT5	hypothetical LOC151162	Cytoplasm	enzyme	•	•	1.112	3.00E-03
PECAM1	platelet/endothelial cell adhesion molecule	Plasma Membrane	other	•	•	2.404	1.01E-04
RBP4	retinol binding protein 4, plasma	Extracellular Space	transporter	•		1.872	2.70E-02
RECK	reversion-inducing-cysteine-rich protein	Plasma Membrane	other	•		2.569	4.60E-02
SELENBP1	selenium binding protein 1	Cytoplasm	other	•		1.327	1.20E-02
SLIT2	slit homolog 2 (Drosophila)	Extracellular Space	other	•		1.848	3.60E-02

Black circle represents the presence in the biofluid.

### Complement factor B (*CFB*)

*CFB*, encodes complement factor B, a component of the alternative pathway of complement activation. Factor B circulates in the blood as a single chain polypeptide. In our study, *CFB* has been retrieved as a potential marker upregulated in urine (fold change, 3.231; rank as No. 2 among 19 entities) for the diagnosis of PCa. Indeed, there have been various studies confirming the important role of *CFB* in PCa. For example, Sardana et al [23] have identified Complement factor B preproprotein as the third most abundant protein in the serum sample of PCa patients. This might implicate the close association of *CFB* with the pathogenesis of PCa. Therefore, the usefulness of *CFB* in the urine detection of PCa is well worth of further investigation.

### Apolipoprotein D (*APOD*/*Apo-D*)

*APOD*, encodes a component of high density glycoprotein which is closely associated with cholesterol acyltransferase, an enzyme involved in lipoprotein metabolism. *APOD* is also involved in the transport and binding of bilin. In our study, *APOD* has been retrieved as a potential marker upregulated in urine (fold change, 2.803; rank as No. 3 among 19 entities) for the diagnosis of PCa. Indeed, there have been various clinical studies confirming the presence of *APOD* in urine and its role in disease detection. For example, Kentsis et al. have discovered *APOD* as one of the putative urine markers for the clinical diagnosis of acute appendicitis [24]. The protein abundance level of *APOD* in urine and its correlation with severity of appendicitis are validated by targeted mass spectrometry. Furthermore, Aspinall et al. has found that elevated *Apo-D* level is closely associated with the advancement of PCa [25]. Put together, *APOD* could be a very promising urine marker for the clinical detection and prognosis of human PCa.

### Reversion-inducing-cysteine-rich protein with kazal motifs (*RECK*)

Extracelluar matrix remodeling is a prerequisite in tumor invasion and often leads to the overexpression of matrix metalloproteinases (MMPs). *RECK* is an inhibitor of MMPs by negatively regulating MMP-2, MMP-9 and MMP14/MT1-MMP activity [26]. Regarding PCa, the role of *RECK* has not yet been clarified. Interestingly, we have retrieved *RECK* as a potential marker upregulated in urine of PCa patients (fold change, 2.569; rank as No. 4 among 19 entities). This might be a reflection of the interrelationship of *RECK* with MMP-2 and MMP-9 along the metastasis process of PCa. Thereby, the potential of *RECK* in the diagnosis/prognosis of PCa has emerged from our study.

### Platelet endothelial cell adhesion molecule (*PECAM1/CD31*)

*PECAM1* is mostly found on the surface of platelets, monocytes, neutrophils, and some types of T-cells. *PECAM1* is known for its key role in removing aged neutrophils from the body [27]. In an early study, Huss et al. [28] have found that *PECAM1* is associated with the early event of angiogenesis and the initiation and progression PCa. This is consistent with our study that *PECAM1* functions as a hub entity in the network of PCa progression and metastasis according to its centrality in the network. We have identified *PECAM1* as a potential upregulated entity in urine of PCa patients (fold change, 2.404; rank as No. 5 among 19 entities). Collectively, *PECAM1* might be explored as a potential urine marker for the diagnosis/prognosis of PCa.

## Discussion

In the present study, we have proposed an integrative mining approach for the identification of putative urine markers specific for PCa detection derived from public genomic profiles. The uniqueness of our approach is that genes specifically overexpressed in PCa were first identified by comparison analysis between PCa, bladder cancer and renal cancer, the three major malignancies in human urinary system. Only in this way, urine markers that are likely to be highly discerning for PCa can be identified by excluding those urinary proteins released in the disease conditions of bladder tumor and renal tumor. The set of controlled GO terms enriched in urine proteome was used as ontology filters in our study to identify genes encoding putative urinary proteins. Indeed, this ontology-based filtering strategy has been frequently used in the mining of functional genomic profiles to derive targets with biological significance. Moreover, to ascertain their presence in urine, these putative PCa urinary proteins were further consulted in databases warehousing urinary proteome data. The pathway enrichment analysis was also adopted in our strategy to investigate the association of the derived entities with the pathogenesis of human cancer. We believe that the assembling of functional genomic data, ontology-based filters, urinary proteome databases and pathway enrichment analysis could be well suited in the discovery of candidate biomarkers in biofluids for the detection of PCa as well as for many other human diseases.

One interesting finding from our study is that a large portion of the derived entities (9 out of 19 entities, see **Table 1**) are present in both urine and blood. This is probably because the quantity of the urine excretion bears a direct proportion of the blood. These markers might be secreted to blood from cancerous cells first and then excreted into urine. Therefore, these entities might be used as candidate markers for PCa screening detectable in both fluids. Another interesting finding from the pathway enrichment analysis is that numerous markers derived from the study are involved in the body's innate immune response system. For instance, *CFB*, *CFH* and *FTL* are found to be associated with the acute-phase signaling pathway, which consists of a large number of proteins produced in response to body inflammation. Hence, these urinary proteins could serve as PCa markers to inform disease progression or disease management with regards to the host defense response of the patients, during which the innate immune system is triggered to attack the tumor cells. Moreover, *MGAT5*, identified as a urine marker for PCa detection, was found to be involved in N-glycan biosynthesis pathway which is crucial in the adhesive or migratory behavior of cancerous cells. Consequently, *MGAT5* could be further investigated as a prognostic marker for PCa invasion and progression. Intriguingly, we have found a few entities (*CRABP1*, *FTL*, *MGAT5* and *SELEBP1*) as putative urine markers annotated by GO terms indicating their intracellular location. This could be accounted by the secretion of intracellular proteins inside small-membrane vesicles named as exosomes released into urine from prostate. Furthermore, cancerous cells undergoing apoptosis are likely to release intracellular matter into urine.

**Figure 5:**
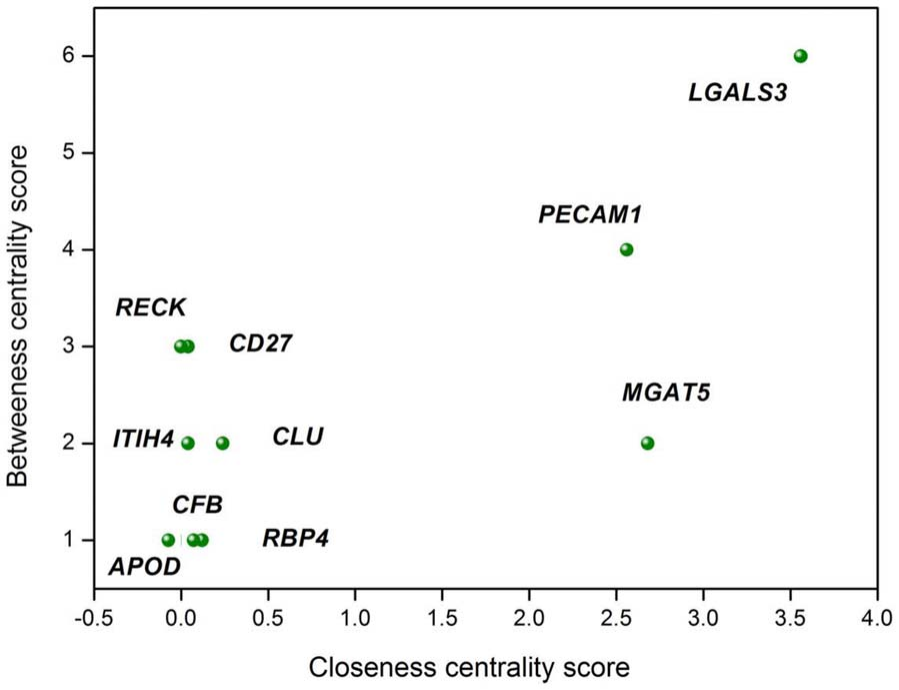
Betweenness centrality score and closeness centrality score for the 10 entities involved in the network of PCa progression and metastasis. Betweeness centrality score was denoted as BC and closeness centrality score was denoted as CC: *LGALS3* (BC, 6.00; CC, 3.56); *PECAM1* (BC, 4.00; CC, 2.56); *CD27* (BC, 3.00; CC, 0.04); *RECK* (BC, 3.00; CC, 0.00); *MGAT5* (BC, 4.00; CC, 2.56); *CLU* (BC, 2.00; CC, 0.24); *ITIH4* (BC, 2.00; CC, 0.04); *RBP4* (BC, 1.00; CC, 0.12); *APOD* (BC, 1.00; CC, 0.00); *CFB* (BC, 1.00; CC, 0.00).

Another merit of our integrative mining approach is the ability to identify ‘focused’ protein interaction networks consisting of derived entities associated with pathogenesis of human cancer. It has been recognized that genes/proteins with potentials as diagnostic or therapeutic targets are more likely to function as a cooperative group or network in human cancer [29]. As an example, we have identified 10 entities (see **Figure 4**) implicated in the process of tumor cell growth, development and proliferation using pathway enrichment analysis. Using these entities as seeds, we have constructed a protein-protein interaction subnetwork which might lead to PCa progression and metastasis. Among the ten entities, we have found that *LGALS3*, *PECAM1*, *MGAT5*, *RECK* and *CLU* function as ‘hub’ entities with high connectivity (a large number of interactions with other entities) in the network. To prioritize the ‘highly influential’ entities in the PCa network, we have applied the concept of graph theory [30] to calculate the betweeness centrality and closeness centrality for each entity. The formulas to calculate the betweeness centrality and closeness centrality are as below,

For a graph *G*: = (*V*, *E*) with *n* vertices, the betweenness centrality *C_B_*(*v*) for vertex *v* is:
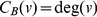
(1)The closeness centrality is defined as the mean geodesic distance (i.e., the shortest path) between a vertex *v* and all other vertices reachable from it,
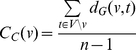
(2)By calculating centrality score for each entity in the PCa network (see **Figure 5**), we have found that *LGALS3*, *PECAM1* and *MGAT5* appear to be the three most ‘influential’ entities in the network with the highest centrality scores. They might be used as prioritized prognostic agents for the detection of PCa invasion and progression. Therefore, strategies could be formulated in the process of PCa treatment to monitor the synergic expression of these entities in urine as strong indicators of therapeutic response and outcome. Moreover, understanding of such networks with related genetic changes which promote tumorigenesis will improve PCa detection and potentially identify novel points of therapeutic intervention.

We recognize several caveats in our mining strategy. First, we make the assumption in the approach that the expression level of a gene is a true reflection of its encoding protein level in the urine. This assumption doesn't always hold true as we mentioned earlier in the paper. Second, the study is limited by the quantity and quality of microarray datasets for the three tumor types. Therefore, the specificity of the PCa urine markers in our study is subject to the availability of the microarray data for each disease. Nevertheless, we believe that our strategy has captured the most important features in the mining of cancer genomics profiles for the discovery of putative markers in body fluids. Further, our strategy is simple to implement for experimentalist and could be used to provide interesting candidate markers for the discovery of clinically useful markers through targeted proteomic analysis.

### Conclusion

We have described herein an integrative and experimentalist-friendly approach to derive potential urine markers for the specific detection of PCa by assembling of cancer gene expression profiles, ontology-based filters, urinary proteome databases and pathway knowledgebase. The application of this strategy has led to the identification of 19 upregulated entities encoding putative urinary protein markers for noninvasive PCa detection. To the best of our knowledge, our study is the first to identify those putative urine markers specific for PCa by comparison analysis across three major tissue types within human urinary system. In addition, our approach offers the advantage of prioritizing candidate markers to detect the invasion and progression of PCa by constructing ‘focused’ interaction subnetworks derived from pathway enrichment analysis. Moreover, these retrieved entities could be used to extract biological insights for dissecting the pathogenesis of human PCa. In general, this integrative mining approach could be broadly applied to discover candidate markers present in body fluids for the diagnosis or prognosis of many other human diseases.

## Supporting Information

File S1 **C# source code and its GUI program to compare entities derived from different tissue types (use CSV files from Microsoft Excel program as inputs).**
(RAR)
